# Oncology Medical Training and Practice: Managing Jordan’s Brain Drain Through Brain Train—The King Hussein Cancer Center Experience

**DOI:** 10.1200/GO.20.00141

**Published:** 2020-07-08

**Authors:** Hikmat Abdel-Razeq, Maha Barbar, Omar Shamieh, Asem Mansour

**Affiliations:** ^1^ King Hussein Cancer Center, Amman, Jordan; ^2^ School of Medicine, University of Jordan, Amman, Jordan; ^3^ Department of Pediatrics, King Hussein Cancer Center, Amman, Jordan; ^4^ Department of Palliative Care, King Hussein Cancer Center, Amman, Jordan

## Abstract

**PURPOSE:**

The medical education system in Jordan is one of the most advanced education systems in the Middle East. Yet many medical school graduates leave the country to seek specialty and subspecialty education and training abroad, and the majority of graduates continue their careers there.

**METHODS:**

We explored reasons behind this so-called “brain drain” and how to slow it, along with capacity building opportunities and strategies for better local training.

**RESULTS:**

By taking advantage of various international collaborative opportunities, the King Hussein Cancer Center has managed to offer strong local training programs and an enhanced working environment, which has enabled us to improve the educational level of our graduates so they can help staff the Center, the country, and the region.

**CONCLUSION:**

Strong local training programs coupled with international partnerships can result in better training for physicians and offset the problem of brain drain without putting any restraints on the graduates.

## INTRODUCTION

Jordan is located in the heart of the Middle East, and the population has surged in the past three decades (after the Gulf War and the Syrian conflict) from 3.5 million in 1990 to 10.8 million in 2020; 91.5% of the population lives in urban areas. With a median age of 23.5 years, the population of Jordan is younger than that of most countries; 61.7% of the citizens are between the ages of 51 and 64 years.^1,2^ Average life expectancy at birth is 75.5 years (77.1 years for females), and the literacy rate, as reported by the United Nations Educational, Scientific and Cultural Organization, was 98.2% in 2018 for adults older than age 15 years.^3^ Jordan is an upper middle–income country; in 2019, the gross domestic product (GDP) was estimated at $44.4 billion, with an annual growth rate of 2.0%.^4^ The health care sector constituted approximately 7.5% of GDP expenditures.^5^

**CONTEXT**

**Key Objective**
This article addresses medical education and training in Jordan, an upper middle–income country, and discusses active programs to help prepare highly qualified graduates in the field of cancer.
**Knowledge Generated**
We present some of the key economic and health indicators in the country and address postgraduate medical education and training. Problems that may lead to brain drain and lack of opportunities to scale up our training are addressed.
**Relevance**
Given the increasing number of patients with cancer in countries like ours, demand is increasing for highly qualified health care workers in the field of cancer. We believe our experience may help low- and middle-income countries scale up their training and education programs to counterbalance the brain drain without putting restraints on their graduates.

### Medical Education in Jordan

The medical education programs in Jordan are considered to be among the strongest in the region. The 6 medical schools in Jordan had 2,500 graduates in 2019. It is estimated that 36,000 physicians have registered with the Jordan Medical Association since its inception in 1954. Currently, around 24,000 physicians are working in Jordan, and more than 4,000 are working abroad. The Jordan Medical Council was established under the umbrella of the Ministry of Health to advance continuing medical education, regulate specialty and subspecialty training, and offer certifying examinations in almost all medical specialties. Jordanian Board Certifications are granted for qualified applicants who pass the designated examinations. Physicians who completed structured training programs abroad have to sit for the Jordanian Board examinations, even if they have certifications from foreign countries.

### Health Care System and Cancer Care in Jordan

The health care system in Jordan is divided between the public and private sectors. There are 41 public and 65 private hospitals with 1.8 beds per 1,000 population.^6^ More than 70% of Jordanians have health insurance, 80% of those with insurance provided by the government.^7^ Jordan is known for medical tourism and attracts more than 250,000 patients annually from neighboring countries that come to Jordan for treatment of several medical problems, including cancer.^8^

Cancer is the second leading cause of death after cardiovascular disease,^9^ and the number of cases is increasing; a total of 10,898 new cases were reported in 2018.^10^ Cancer care is totally covered by the government for all citizens through Jordan’s pub-lic hospitals. This coverage includes chemotherapy, monoclonal antibodies, and some of the recently approved immunotherapy agents, the availability of which depends on cost-effectiveness studies. King Hussein Cancer Center (KHCC) is a 352-bed, stand-alone cancer center that was established almost 20 years ago. It is the most comprehensive tertiary cancer center in Jordan and the region. In 2019, KHCC treated more than 6,000 new patients with cancer, had 250,000 outpatient clinic visits, delivered 45,000 chemotherapy sessions, and had almost 15,000 admissions. The center is accredited by the Joint Commission International,^11^ College of American Pathologists,^12^ MAGNET,^13^ and Accreditation of Human Research Protection Programs.^14^

### Problems With Staffing

Since its establishment, the KHCC recognized that staffing the center with highly qualified health care workers would be a challenge. The center currently employs more than 250 full-time consultants and specialists, 109 residents, and 38 fellows in medical, pediatric, surgical, and radiation oncology. Higher income offered by many neighboring Gulf countries used to be the main driver for many physicians, mostly younger ones, to move out of the country, but lately, this problem has decreased somewhat. Still, hundreds of our very ambitious young and healthy new medical graduates are leaving the country annually to seek specialty and subspecialty training in Western countries. The United States of America, the United Kingdom, Germany, and Australia are among the top countries that attract medical school graduates. Unfortunately, the majority of recently trained physicians do not come back to Jordan.^15^ Even the minority (less than 10%) who choose to come back to Jordan soon return to the countries where they received their specialty training.

Jordan is not alone; this is worldwide problem. Physicians migrate to Western countries for lots of reasons^16^: higher-quality medical education, better quality of life, high level of medical technology, better research opportunities, better school systems, and higher wages in the destination countries.^17^ Gaps are getting wider in health care delivery, training, education, and research output between low- and middle-income countries (LMICs) and high-income countries (HICs).

### No Restraints

We recognized the phenomenon of so-called “brain drain” early on. We also agree that our country should put no restraints on the ambitious physicians who seek to advance their career development plans and invest in opportunities outside Jordan. We at KHCC have taken many steps to help them do so. Transitional residency training programs in internal medicine, pediatrics, and surgery were established a long time ago. Applicants often spend a transitional year or two getting hands-on training and clinical practice in a setup that matches that of the best hospitals in West-ern countries. During this time, applicants prepare for and finish the entry examinations mandated by Western accredited structured training programs, such as the United States Medical Licensing Examination, the Professional and Linguistic Assessment Board examination in the United Kingdom, and the Fachsprachenprüfung medical language examination in Germany. Passing these examinations enables hundreds of physicians to secure positions in the most reputable training centers.

At the same time, we believe that such physicians when finished with their training abroad should create a plan to pay back their own countries. Many have already started to organize rotating voluntary work in their home countries to improve the delivery of specialized clinical care and help scale up training, education, and networking for research. They are using advanced technology like TeleSynergy and Web meetings for remote consultation, multidisciplinary case discussion, delivery of training and education, and collaborative research projects. Other options include transferring knowledge and skills to home countries by mentorship and shared publications.

### Training Programs

#### Structured oncology training programs.

At KHCC, we took an additional approach that paid off and continues to pay off because it enabled us to run the biggest and busiest cancer center in the region. We have established strong structured training programs in almost all cancer-related specialties, including residency programs in radiation oncology and fellowship training programs in both pediatric and adult medical and surgical oncology and more recently in palliative care. All are accredited by the Jordanian Medical Council, and Board Certifications are offered in these specialties. In addition, we have combined our training programs in radiology, cancer imaging, breast imaging, nuclear medicine, pathology, and anesthesia with those of many local hospitals. Mentors and directors of such programs were trained mostly in United States and the United Kingdom and have experience with the training requirements and curriculums in Western countries. During their last year of training, fellows are encouraged to perform 2 to 3 months of fully sponsored elective rotations at reputable institutions in the United States, Canada, or Europe. Most of the training graduates are given competitive offers by KHCC or local hospitals.

#### In-training examinations.

To strengthen our training programs, we mandate that our trainees sit for the annual in-training examination in radiation oncology and medical oncology provided by the American College of Radiology^18^ and ASCO,^19^ respectively. Results of such examinations are used to modify and improve our training programs. Performance on such examinations has always been reassuring, and it is interesting to mention here that our center was ranked at the top of all North American fellowship programs in the latest 2019 ASCO examination.

#### Postgraduate subspecialty training.

One of the most rewarding steps we have taken was to team up with many reputable centers like the Princess Margaret Hospital in Toronto, Ontario, Canada; the MD Anderson Cancer Center in Houston, Texas; and the Leeds Cancer Centre-St. James’s Institute of Oncology in Leeds, United Kingdom. These agreements enabled many of our young medical, surgical, and radiation oncologists, after completing their local training and graduation, to join these centers for sponsored subspecialty training for 1 or 2 years. Many reputable cancer centers around the world welcome collaborations with counterparts in LMICs; communications and leadership networking are key factors here. This shorter-than-the-usual mandatory 3-year structured fellowship program and the lack of initial board certifications in internal medicine, surgery, or pediatrics will not allow applicants to get the medical licenses they need to practice and stay abroad, and thus almost all of them return home. On the rare occasions when fellows elected not to return to Jordan, they were asked to repay all stipends and associated costs paid by the center. Depending on the needs, 8 to 10 fellows are sponsored by our institution annually.

#### Career development.

As part of the training process, fellows are prepared by their mentors and directors for post-fellowship careers; a clear plan for professional development is usually drawn up and addressed with each fellow while they are in training.^20^ After their graduation and appointment to the hospital staff, graduates are given the same responsibilities, duties, and titles as those with board certifications from Western countries. Since we started, more than 100 adult and pediatric oncologists in different specialties have graduated; some are serving as consultants at our center and local institutions, and many others are currently practicing in neighboring Gulf countries. In addition, many non-Jordanian trainees have returned to serve their own people in neighboring countries, including Iraq, Yemen, Palestine, Bahrain, Sudan, Oman, and Libya. In addition to their specialty clinical practice in medical oncology, radiation oncology, pathology, bone marrow transplantation, and palliative care, many have taken leadership roles to promote cancer care in their own countries.

#### Beyond Clinical Training

A new residency program in medical physics, the first in the region, is a 2-year program that was established at KHCC in collaboration with a local Jordanian university and is supported by the International Atomic Energy Agency. Another 2-year pharmacy residency program was established by the Department of Pharmacy at KHCC. It has 6 core modules, including general pharmacy operation, drug information, adult internal medicine, pediatrics, ambulatory care, and intensive care. Elective rotations in bone marrow transplantation, surgery, palliative care, pharmaco-economics, health care quality, and patient safety are also offered. The world’s first master of cancer informatics degree program is a joint program between KHCC, the University of Jordan, and the University of West England in Bristol, United Kingdom.

It is important to highlight that HICs that benefit the most from Jordan’s brain drain, such as the United States, the United Kingdom, Canada, and Australia should take responsibility for their ethical obligation to repay LMICs, where some of their valuable immigrant physicians have come from. A merit-based immigration plan announced by the US government might make it easier for highly skilled individuals from LMICs to move to the United States and thus worsen the already serious problem of brain drain.^15^

Providing opportunities to train physicians of LMICs who opt to stay in their home countries is the least they can offer, and this should be addressed at the governmental level. ASCO took some steps in this regard. One example is the establishment of the International Development and Education Awards for early-career oncologists in LMICs. Recipients of this award receive support to attend the ASCO Annual Meeting and are paired with ASCO members in institutions in the United States or Canada.^21^ ASCO also offers a long-term international fellowship or a 1-year leadership development program.^22^ Many similar opportunities are also available through professional societies and organizations in Europe. The European School of Oncology offers 3- to 6-month fellowship programs in specific disciplines, such as surgical oncology, pediatric oncology, gynecologic oncology, or organ-specific cancer (eg, lung cancer). These programs encourage applicants from Arab countries to apply.^23^ The Union for International Cancer Control offers 1- to 3-month technical fellowship programs.^24^ However, most of these programs tend to be short rotations, and they are available to only a few applicants, so they have had no major impact on staffing levels in LMICs.

#### Local Obstacles

Low income and political instabilities in neighboring countries are just 2 of many obstacles that Jordan faces in retaining its medical graduates. Local` legislation in our country is not helping, either. Many highly trained physicians who studied abroad, even those with board certifications from Western countries, find it too difficult to pass local board certification examinations. Some progress had been made in this regard, and many physicians who finished their training before 2001 were granted reciprocal Jordanian Board Certification without taking the examination. More serious efforts are needed by local governmental agencies to solve this long-standing problem, which could be one of the reasons that prevent some of the younger generation of physicians to at least try a career in their home country.

#### Future Directions

KHCC is looking to strengthen its goal to become a hub for cancer-related training and education that will graduate physicians to serve the country and the region. KHCC is also expanding its base to include accredited training programs in areas such as pediatric palliative care and cancer survivorship. We believe that establishing local high-quality structured training programs that are linked to key performance indicators and sweetened by a year or two of non-board certified subspecialty training at specialized Western institutions will help us scale up our level of training. This will also help us build high-quality collaborative research programs. We believe our plans to offer specialized training will minimize our current problem of brain drain without imposing restraints on our trainees.
